# Health-promoting lifestyle behaviors and its association with sociodemographic characteristics in hospital clinical staff

**DOI:** 10.3389/fpubh.2024.1391094

**Published:** 2024-09-04

**Authors:** Alireza Moghimi, Mohsen Saberi Isfeedvajani, Mohammad Javanbakht, Leila Khedmat

**Affiliations:** ^1^Faculty of Medicine, Department of Community Medicine, Baqiyatullah University of Medical Sciences, Tehran, Iran; ^2^Nephrology and Urology Research Center, Clinical Sciences Institute, Baqiyatallah University of Medical Sciences, Tehran, Iran; ^3^Health Management Research Center, Baqiyatullah University of Medical Sciences, Tehran, Iran

**Keywords:** hospital clinical staff, health, lifestyle, behaviors, patients

## Abstract

**Objective:**

The aim of this study was to determine hospital clinical staff’ health-promoting lifestyle behaviors, and explore associations between nurse demographic factors and lifestyle behaviors.

**Methods:**

This cross-sectional investigation focused on the clinical personnel employed at hospitals associated with Baqiyatullah University. A sample of 341 clinical staff of hospitals was collected using convenience sampling. In this study, the questionnaire of Health Promoting Lifestyle Profile II (HPLP-II) was used to assess health-promoting behaviors.

**Results:**

In the present study, the mean HPLP score was 131 ± 23. The score of health-promoting behaviors was significantly higher in the nursing major (*p* = 0.029). Also, a difference was found between the major and the subscales of health responsibility (*p* = 0.000), stress management (*p* = 0.004), physical activity (*p* = 0.004) and nutrition (*p* = 0.001). The score of health responsibility, stress management, physical activity and nutrition subscales was higher in nursing. There was a significant relationship between education and stress management (*p* = 0.033) and physical activity subscales (*p* = 0.001). The physical activity score was also higher in individuals with master’s and doctoral degrees, and the stress management score was higher in participants with master’s degrees. Based on the findings presented herein, age (*p* = 0.001) and gender (*p* = 0.016) were associated with the nutrition subscale, and the nutrition score was higher in the age group of over 30 years and in women. Additionally, a significant relationship was observed between marriage and the subscales of spiritual growth (*p* = 0.013) and nutrition (*p* = 0.024), and the score of spiritual growth, and nutrition was higher in married people. There was a significant relationship between job and health responsibility (*p* = 0.013) and nutrition (*p* = 0.022), and the score of health responsibility and nutrition score was found to be higher in nurses.

**Conclusion:**

Health-promoting behaviors of hospital employees are at an average level and are related to the educational levels of the employees, so these behaviors are more in nurses, while this relationship was not present in physicians. These findings may be helpful in providing recommendations for developing healthy lifestyle programs for clinical staff aimed at promoting health behaviors.

## Background

Growing evidence supports the idea that individuals can actively improve their well-being (1). Health Promotion (HP) is a key strategy for fostering global health, defined by the WHO as enabling people to improve their health (2). There’s a significant emphasis on Health Promoting Lifestyle Behaviors (HPLB) in recent years, encompassing actions and beliefs across various aspects of health promotion to elevate well-being and reduce illness, including responsibility, physical activity, nutrition, interpersonal relations, self-actualization, and stress management are of great importance for the optimal health and well-being (3).

Various studies across different societies have delved into the status of Health-Promoting Behaviors and the factors that impact them. Factors such as age, gender, health status, education level, and marital status have consistently emerged as influences on health-promoting behaviors, as evidenced by research findings (4–6).

Clinical staff are involved in health-promoting behaviors, where their healthy behaviors can be of great importance (4, 7). Despite the movement to promote health-promoting behaviors among the general public, the health behaviors of nurses, physicians, and community health workers need to be improved to promote physical, social and psychological wellbeing. It has been found that nurses neglect their health promotion role and do not engage in healthy lifestyle behaviors compared to other healthcare professionals due to the nature of their job (8–11), which involves irregular work schedules, long working hours and other work-related stressors (12). Such work-related stressors may lead to the use of ineffective mechanisms such as decreased physical activity, poor dietary choices, overeating, and smoking (13, 14). Employing holistic approaches and/or spiritual health practices increases the possibility of seeing and caring for oneself as a whole—mind, body, emotion, and spirit (14).

Health program interventions for hospital employees such as nurses can help to cope with stress, fatigue, and increase physical health (14) and prevent chronic diseases by promoting healthy lifestyle behaviors and strengthening their flexibility.

Therefore, the current study aimed at exploring the frequency of factors affecting health-promoting behaviors among clinical staff working in Baqiyatallah Hospital in Tehran city, Iran.

## Materials and methods

### Subjects

This cross-sectional study involved clinical staff from hospitals associated with Baqiyatullah University, specifically those working in the CCU, ICU, operating room, emergency room, and both internal medicine and surgery departments. A sample of 341 clinical staff of hospitals was collected using convenience sampling from April 1^st^, 2023 to July 30^th^, 2023. There were no exclusion criteria for individuals, only questionnaires with more than 10% of questions unanswered were excluded from the study. The minimum required sample size was 282 under the assumptions of a mean and standard deviation of the health promotion behavior score (113.08 ± 25.67), a D of 3, and a significant level of 0.05.

### Measures

In this study, the questionnaire of Health Promoting Lifestyle Profile II (HPLP-II) was used to assess health-promoting behaviors. This questionnaire has 52 items across six subdomains and its purpose is to measure health-promoting behaviors (nutrition, health responsibility, physical activity, spiritual growth, stress management, and interpersonal relations). Its response range is of Likert type, and the score for each option is presented with “never” (1 point), “sometimes” (2 points), “often” (3 points), and “routinely” (4 points). Higher mean scores are considered higher engagement in health-promoting behaviors.

### Statistical analysis

Data were analyzed using SPSS Statistics software (version 22.0). Descriptive indices (mean, standard deviation) were applied to characterize the sample. Differences in health-promoting behaviors were assessed using the Mann–Whitney test, and the Kolmogorov–Smirnov test was employed to examine the normality of quantitative data. *p-values* < 0.05 were considered statistically significant.

## Results

### Descriptive statistics

Questionnaire data from 341 clinical staff were analyzed. People were in the age range of 19 to 63 years with a mean age of 34.3 ± 9.4 years (median 31 years). The demographic information of the participants is presented in Table 1. The majority of the study population included female employees (51%) with bachelor’s degrees (38.7%) and nursing education (71.8%). About 68.6% of people were married and 71.8% had a nursing job. Most of the study population had no underlying disease (93%) and did not smoke (95.3%).

**Table 1:** Demographic information of clinical staff.

Variable	Level	Frequency	Percent
Age	Less than or equal to 30 years	161	47.2
	More than 30 years	180	52.8
Gender	Male	174	51
	Female	167	49
Education	Internet	63	18.5
	Associate Degree	24	7
	Bachelor’s degree	132	38.7
	resident	26	7.6
	Master’s degree	75	22
	Expert	7	2.1
	Doctorate	14	4.1
Field of Study	nursing	245	71.8
	medical	96	28.2
Marital status	Single	103	30.2
	Married	234	68.6
	Widow	4	1.2
Job	General medicine student - Interne	63	18.5
	Specialization student-resident	26	7.6
	Nurse	245	71.8
	Doctor	7	2.1
Workplace section	CCU	16	4.7
	ICU	48	14.1
	Surgery/operating room	72	21.1
	Emergency	31	9.1
	Internal	71	20.8
	Other	103	30.2
Underlying disease	no	317	93
	Yes	24	7
Smoking	no	325	95.3
	Yes	16	4.7

### Overall score and mean score of health-promoting behaviors of clinical staff

Based on the Kolmogorov test, the distribution of the total score of the questionnaire and its dimensions was normal (*Z* = 1.096, *p* = 0.181). Considering that the questionnaire has 52 questions and is on a Likert scale of 1 to 4, the total HPLP score was between 52 and 208. In our research, the mean HPLP score was 131 ± 23. According to the general score range of the questionnaire, the score of the health promoting behaviors of the employees was at an average level. According to Table 2, the highest mean score was related to spiritual growth (24.4 ± 4.8) and the lowest score was related to physical activity (17.6 ± 5.5).

**Table 2:** Mean total score and dimensions of health promoting behaviors in clinical staff.

Dimensions	Minimum score	Maximum score	Average	Standard deviation
Spiritual growth	10	36	24.39	4.87
Responsibility for health	11	36	22.55	4.83
Interpersonal support	12	36	24.02	4.25
Stress management	9	32	18.87	4.43
Physical activity	8	32	17.62	5.59
Nutrition	11	36	23.66	4.46
Total score	74	208	131	23.14

### Correlation of the score of health promoting behaviors with demographic characteristics

The results of the Mann–Whitney test showed that the Mean score of the questionnaire did not differ significantly between age groups (*p* = 0.998). In examining the dimensions of health-promoting behaviors, there was a significant relationship between age and nutrition factor (*p* = 0.001). The mean/median nutrition score was higher in the age group over 30 years old [The Mean score of the questionnaire was not significantly different between the two sexes (*p* = 0.923)]. In examining the dimensions of health-promoting behaviors, there was a significant relationship between gender and nutrition subscale (*p* = 0.016). The mean/median nutrition score was higher in women (Table 3).

**Table 3:** Mean/median total score and dimensions of health promoting behaviors of clinical staff according to age.

Age		Spiritual growth and self-actualization	Responsibility for health	Interpersonal support	Stress management	Exercise and physical activity	Nutrition	Total score
≤30	Average	24.30	22.35	24.2	18.85	17.69	22.85	130.55
	Standard deviation	4.92	4.74	4.39	4.28	5.12	4.49	223.9
	Median	24	22	24	18	17	23	131
≥30	Average	24.4	22.7	23.85	18.8	17.56	24.3	131.3
	Standard deviation	4.84	4.91	4.118	4.58	5.99	4.32	23.8
	Median	24	22	24	19	17	24	127
*P*-value		0.676	0.551	0.452	0.884	0.696	**0.001**	0.998

The Mean score of the questionnaire was not found to be significantly different between the two sexes (*p* = 0.923). In examining (*p* = 0.016). The mean/median nutrition score was found to be higher in women (Table 4).

**Table 4:** The mean/median of the total score and dimensions of clinical staff’s health promoting behaviors according to gender.

Gender		Spiritual growth and self-actualization	Responsibility for health	Interpersonal support	Stress management	Exercise and physical activity	Nutrition	Total score
Female	Average	24.3	23.01	24.19	18.89	17.51	24.26	131.6
	Standard deviation	5.06	4.804	4.54	4.62	5.83	4.58	24.7
	Median	24	22.5	24	18	17	24	128
Male	Average	24.44	22.08	23.84	18.86	17.74	23.03	130.3
	Standard deviation	4.67	4.83	3.94	4.25	5.35	4.25	21.4
	Median	24	22	24	19	17	23	129
*P*-value		0.803	0.096	0.643	0.732	0.566	**0.016**	0.923

The results of the Kruskal-Wallis test show (Table 5) that the Mean score of the questionnaire does not differ significantly between different levels of education (*p* = 0.143). A significant relationship was revealed between education and stress management factors (*p* = 0.033) and physical activity (*p* = 0.001) when dimensions of health-promoting behaviors were assessed. The mean/median score of physical activity was higher for master and doctoral degree. The mean/median stress management score was higher in individuals with master’s degree.

**Table 5:** Mean/median total score and dimensions of health promoting behaviors according to education.

Education		Spiritual growth and self-actualization	Responsibility for health	Interpersonal support	Stress management	Exercise and physical activity	Nutrition	Total score
Intern	Average	24.05	21.33	24.32	18.03	16.75	22.68	127.51
	Standard deviation	4.80	4.63	4.17	4.19	5.04	4.53	19.77
	Median	23.00	21.00	25.00	17.00	17.00	22.00	124.00
Associate degree	Average	23.17	22.39	22.22	18.46	17.00	22.92	125.41
	Standard deviation	3.83	4.37	3.59	3.08	4.92	4.00	17.25
	Median	24.00	23.00	21.00	19.00	16.00	23.00	123.50
Bachelor’s degree	Average	24.45	22.68	23.66	18.58	17.17	24.03	130.23
	Standard deviation	5.18	4.85	4.43	4.49	5.90	4.53	24.69
	Median	24.00	22.00	23.00	18.00	17.00	24.00	127.50
Resident	Average	24.50	21.46	24.54	18.19	15.46	22.88	127.04
	Standard deviation	3.97	5.11	3.69	4.60	5.02	4.11	20.57
	Median	24.00	21.00	24.00	18.00	14.50	23.00	125.50
Master’s degree	Average	24.55	23.88	24.54	20.36	19.92	24.49	137.77
	Standard deviation	4.90	4.70	4.18	4.54	5.38	4.32	24.23
	Median	24.00	23.50	25.00	20.00	20.00	25.00	136.50
Expert	Average	23.57	21.29	24.17	18.71	16.29	22.00	124.67
	Standard deviation	1.99	2.75	2.56	4.07	4.07	3.00	11.64
	Median	23.00	21.00	24.00	21.00	17.00	22.00	125.50
Doctorate	Average	26.79	22.86	25.21	19.71	19.71	23.79	138.07
	Standard deviation	6.03	6.15	5.32	5.18	5.61	5.59	27.80
	Median	27.00	22.50	25.00	19.00	18.50	24.00	135.50
*P*-value		0.509	0.076	0.153	**0.033**	0.**001**	0.070	0.143

The Kruskal-Wallis test revealed that the Mean score of the questionnaire does not have a significant difference in terms of marital status (*p* = 0.495). The evaluation of dimensions of health-promoting behaviors showed that there is a significant relationship between marriage and factors of spiritual growth (*p* = 0.013) and nutrition (*p* = 0.024). The mean/median of spiritual growth and nutrition scores was higher in married people (Table 6).

**Table 6:** Mean/median total score and dimensions of clinical staff promoting behaviors according to marriage.

Marital status		Spiritual growth and self-actualization	Responsibility for health	Interpersonal support	Stress management	Exercise and physical activity	Nutrition	Total score
Single	Average	23.51	21.87	23.49	18.68	18.04	22.59	128.2
	Standard deviation	4.62	3.98	4.06	3.78	5.22	4.28	19.4
	Median	24	22	24	19	17	22	127
Married	Average	24.84	22.87	24.309	18.96	17.46	24.12	132.3
	Standard deviation	4.93	5.15	4.32	4.71	5.73	4.504	24.6
	Median	25	22	24	18	17	24	130
Widow	Average	20.25	21.25	21	19	16.5	24	122
	Standard deviation	2.06	4.27	2.82	4.24	7.23	1.41	18.6
	Median	20.5	21.5	20	18.5	16.5	24.5	121
*P*-value		**0.013**	0.326	0.094	0.993	0.584	0.**024**	0.495

According to the results of the analysis of variance, no significant difference was found in the overall score of the questionnaire in terms of occupation (*p* = 0.514). In evaluating the dimensions of health-promoting behaviors, there was a significant relationship between occupation and health responsibility (*p* = 0.013) and nutrition (*p* = 0.022). The mean/median score of responsibility regarding health and nutrition score was higher in nurses (Table 7).

**Table 7:** Average/median total score and dimensions of employee health promoting behaviors according to occupation.

Job		Spiritual growth and self-actualization	Responsibility for health	Interpersonal support	Stress management	Exercise and physical activity	Nutrition	Total score
General medicine student - Interne	Average	24.21	21.05	24.64	17.94	16.77	22.72	127.90
	Standard deviation	5.01	4.77	4.23	4.20	5.28	4.60	21.06
	Median	23.00	21.00	25.00	17.00	16.50	22.00	124.00
Specialization student-resident	Average	24.81	21.65	24.32	18.32	15.74	23.65	128.48
	Standard deviation	3.87	5.40	3.80	4.71	5.18	4.23	20.80
	Median	25.00	21.00	24.00	18.00	15.00	25.00	126.00
Nurse	Average	24.37	23.05	23.79	19.17	18.11	24.04	132.12
	Standard deviation	4.96	4.72	4.29	4.46	5.78	4.43	24.04
	Median	24.00	23.00	24.00	19.00	18.00	24.00	131.00
Doctor	Average	23.55	22.82	23.70	19.27	17.64	21.18	127.60
	Standard deviation	5.18	3.06	4.72	4.15	3.53	4.49	22.51
	Median	22.00	22.00	23.00	21.00	18.00	21.00	125.50
*P*-value		0.705	**0.013**	0.440	0.080	0.073	**0.022**	0.514

The results of the Mann–Whitney test showed that there was no significant difference in the overall score of the questionnaire according to smoking (*p* = 0.484). No significant relationship was observed in the examination of dimensions of health-promoting behaviors in terms of smoking (Table 8).

**Table 8:** The mean/median of the total score and dimensions of employee health-promoting behaviors according to smoking.

Smoking		Spiritual growth and self-actualization	Responsibility for health	Interpersonal support	Stress management	Exercise and physical activity	Nutrition	Total score
No smoking	Average	24.34	22.53	24.04	18.79	17.53	23.70	130.70
	Standard deviation	4.83	4.73	4.16	4.37	5.56	4.41	22.76
	Median	24.00	22.00	24.00	18.00	17.00	23.50	128.00
a smoker	Average	25.31	23.13	23.56	20.75	19.73	22.81	135.87
	Standard deviation	5.75	6.72	5.99	5.43	6.05	5.62	30.32
	Median	25.50	24.00	24.00	19.00	20.00	23.00	135.00
*P*-value		0.589	0.670	0.686	0.217	0.150	0.634	0.484

As the results of the Mann–Whitney test showed, the overall score of the questionnaire was significantly different according to the major (*p* = 0.029). The mean/median score of health-promoting behaviors was higher in nursing. In examining the dimensions of health-promoting behaviors, there was a significant relationship between the major and the factors of health responsibility (*p* = 0.000), stress management (*p* = 0.004), physical activity (*p* = 0.004) and nutrition (*p* = 0.001). The mean/median score of responsibility for health, stress management, physical activity and nutrition was also found to be higher in nursing (Table 9).

**Table 9:** Mean/median total score and dimensions of health promoting behaviors of employees according to major.

Field of Study		Spiritual growth and self-actualization	Responsibility for health	Interpersonal support	Stress management	Exercise and physical activity	Nutrition	Total score
Nursing	Average	24.47	23.16	23.86	19.28	18.25	24.16	132.80
	Standard deviation	4.88	4.69	4.23	4.43	5.73	4.41	23.71
	Median	24.00	23.00	24.00	19.00	18.00	24.00	131.00
Medical	Average	24.15	21.13	24.33	17.97	16.37	22.53	126.74
	Standard deviation	4.85	4.78	4.27	4.32	5.12	4.45	21.28
	Median	23.00	21.00	24.00	17.00	16.00	22.00	124.00
*P*-value		0.448	**0.000**	0.324	**0.004**	**0.004**	**0.001**	0.**029**

## Discussion

Although in most of the literature, health promotion has been discussed for students, academic staff and patients, there are very few studies regarding clinical staff. In our study, the mean score of health promoting behaviors in clinical staff was 131 ± 23, which is at an average level.

The mean score for health-promoting behaviors among nursing students from three universities in South Korea has been reported to be 2.47, higher than the midpoint (4). Many studies have observed a medium level of health promotion behavior among health professionals (15–18).

In our study, among the subscale of health-promoting behaviors, the mean score of spiritual growth (24.4 ± 4.8) was at a high level, while physical activity (17.6 ± 5.5) was at a low level.

Many previous studies have reported an association of social-demographic variables such as age, education, income, and health status with subscales of health-promoting behaviors (19).

In the present study, the main factors affecting health-promoting behaviors were age, gender, education, marriage, major, and occupation.

The results of examining the relationship between demographic factors and the overall score of health-promoting behaviors showed that there were significant results related to the major of clinical staff specialists (*p* = 0.029). The score of health-promoting behaviors was higher in nursing, although the total score was at an average level (132.8 ± 23.7).

A significant relationship was found between the subscales of health-promoting behaviors and the nursing major, where the score of health responsibility, stress management, physical activity and nutrition subscales in nursing was higher than that of doctors. Also, our results showed that nursing had the highest score in responsibility and nutrition, and had the lowest score in the areas of stress management and physical activity. This could be explained by nurses’ heightened awareness of the importance of these behaviors due to their training and professional responsibilities, aligning with the health belief model’s emphasis on perceived benefits and barriers influencing health behaviors (20).

In the study of Mahmoodi et al. (15), which compared the health-promoting behaviors of nurses, health and administrative workers, spiritual growth and nutrition were at a high level, and the lowest score was related to physical activity. Estebsari et al. (16) have shown that the highest score was related to spiritual growth in hospital employees in Gilan, Iran, while they scored the lowest in the areas of physical activity and stress management. Another study showed that the mean score of interpersonal support was highest among nursing students from South Korea, and the mean scores of health responsibility and physical activity were the lowest (4). The results of the aforementioned studies are more or less consistent with our study.

It has been found that stress management showed the lowest mean in hospital nurses and interpersonal relationships had the highest mean (21). The difference between the findings of studies may be associated with personal and environmental factors that affect participation in health-promoting behaviors, including past experience, cultural issues, lack of time management, work fatigue, sport equipment and facilities, inappropriate policies for leisure time, working conditions, lack of attention by individuals and authorities to immobility and support or organizational norms (7, 22). Working conditions and the burden of care services can be one of the reasons for low physical activity in nurses. However, identifying factors that hinder health-promoting behaviors and training them may be effective in their control. One of the ways that nursing can move toward professional excellence is to use approaches that encourage people to accept responsibility for their own health, and this importance will undoubtedly be realized by increasing the health responsibility in nurses themselves. Furthermore, just nursing knowledge of the importance of health promotion does not always lead to self-care (4, 23–25).

Other results of our study showed a significant relationship between gender and nutrition subscale, when the nutrition score was higher in women. Larouche (25) expressed that women had significantly better behavior in nutrition, interpersonal relations, and health responsibility compared to men. Another study showed that women scored higher than men in subscales of nutrition among nursing students of Tehran city.

Based on the results presented herein, a significant relationship was found between age and subscale of nutrition (*p* = 0.001), where the nutrition score was higher in the age group of more than 30 years. A study on nursing students has shown that the scores under the subscales of nutrition as well as spiritual growth, health responsibility and stress management were significantly lower in the younger age group than in other age groups (18). Another study reported a significant association between the age of nursing students and the sub-scales of stress management and physical activity (26). Younger nurses demonstrated differences in physical activity, stress management, and health responsibility (27); however, older age of students has been found to be associated with higher levels of overall health-promoting behavior when compared with younger students (28), while other study reported lack of association of age with the total score of HPLP II (29). The theory of planned behavior emphasizes the role of attitudes, subjective norms, and perceived behavioral control in shaping intentions and behaviors (30). The significant associations found between age, gender, marital status, and job type with specific health-promoting behaviors align with this theory. For instance, the higher nutrition scores among women and individuals over 30 years old may be influenced by their attitudes toward healthy eating and perceived behavioral control over their dietary choices. Similarly, the differences in health responsibility and nutrition scores across job types indicate varying social norms and perceived control over health behaviors in different occupational settings.

According to another finding of our study, there was a significant relationship between education with stress management and physical activity, where the score of physical activity was higher in master’s degree and doctoral studies and the score of stress management was higher in participants with master’s degrees. Similar results were reported in Hosseini et al.'s (7) study, when the score of stress management and health responsibility was significantly higher at a higher education level. However, in Chow et al.’s study, which was conducted on 314 s- and fifth-year nursing students, the score of the health responsibility subscale was significantly higher with the increase in the duration of education (31). In a recent study conducted by Hwang et al. (32) on 304 nursing students, the score of responsibility increased with increasing education. Thus, higher education levels may lead to greater self-efficacy and motivation to engage in health-promoting behaviors. Professionals with master’s and doctoral degrees may have acquired more knowledge and skills to effectively manage stress and engage in physical activity, reflecting the role of self-efficacy in behavior change within the social cognitive theory framework (33).

It can be assumed that nursing students with nursing curriculum training on the importance of promoting Health, increase responsibility for their health. It can be assumed that teaching the nursing curriculum about the importance of health promotion increases the responsibility for health in nursing students. However, other factors should not be neglected.

Also, a significant relationship between marriage and subscales of spiritual growth (*p* = 0.013) and nutrition (*p* = 0.024) was seen in the present study when the scores of spiritual growth and nutrition were higher in married people. Hosseini et al.’s study showed that the score of nutrition and spiritual growth subscales were significantly higher in married students than in single students (7). One of the reasons for the difference in the results can be attributed to the smaller sample size in the present study.

The results of our study showed a significant relationship between job and subscales of health responsibility (*p* = 0.013) and nutrition (*p* = 0.022), and the score of health responsibility and nutrition was higher in nurses.

In Mahmoodi et al.’s (15) study, there was a significant relationship between the type of job and the subscales of responsibility, spiritual growth, stress management, and nutrition, where nurses had a more favorable situation compared to administrative staff. Tsai and Liu (32) evaluated factors associated with health-promoting behaviors among hospital staff in Taiwan, where health responsibility and interpersonal support have been reported to be positively linked to work experience, while stress management, spiritual growth, physical activity, and nutrition were negatively associated with daily work times. Work experience is considered as a more important factor in this context, stress management was significantly higher in-hospital staff with work experience of more than 10 years (16). One of the reasons for the difference in results can be attributed to the difference in sample size.

## Conclusion

The results of the present study demonstrated that the health-promoting behaviors of hospital employees are at an average level and are related to the educational levels of the employees, so these behaviors are more in nurses, while this relationship was not present in doctors. Also, the score of health responsibility, stress management, physical activity and nutrition subscales was found to be higher in the major of nursing. Regarding the lower score of physical activity and stress management, it seems that these two areas should be considered more by nurses. In addition, the factors of stress management and physical activity were influenced by the level of education. The nutrition subscale was related to age, gender and marriage. The spiritual growth subscale was influenced by the married status, and the health responsibility subscale was also significantly associated with the employees’ jobs.

## Data Availability

The dataset generated and analyzed during the study is available from the corresponding author on reasonable request.
